# Associations of Lifestyle Factors, Disease History and Awareness with Health-Related Quality of Life in a Thai Population

**DOI:** 10.1371/journal.pone.0049921

**Published:** 2012-11-26

**Authors:** Prin Vathesatogkit, Piyamitr Sritara, Merel Kimman, Bunlue Hengprasith, Tai E-Shyong, Hwee-Lin Wee, Mark Woodward

**Affiliations:** 1 The George Institute for Global Health, University of Sydney, Sydney, New South Wales, Australia; 2 Department of Medicine, Faculty of Medicine Ramathibodi Hospital, Mahidol University, Bangkok, Thailand; 3 Medical and Health Office, Electricity Generating Authority of Thailand, Nonthaburi, Thailand; 4 Department of Medicine, National University Health System, Singapore, Singapore; 5 Saw Swee Hock School of Public Health, National University of Singapore, Singapore, Singapore; 6 Department of Pharmacy, National University of Singapore, Singapore, Singapore; Mayo Clinic College of Medicine, United States of America

## Abstract

**Background:**

The impact of the presence and awareness of individual health states on quality of life (HRQoL) is often documented. However, the impacts of different health states have rarely been compared amongst each other, whilst quality of life data from Asia are relatively sparse. We examined and compared the effects of different health states on quality of life in a Thai population.

**Methods:**

In 2008–2009, 5,915 corporate employees were invited to participate in a survey where HRQoL was measured by the Short Form 36 (SF-36) questionnaire. The adjusted mean SF-36 scores were calculated for each self-reported illness, number of chronic conditions, lifestyle factors and awareness of diabetes and hypertension. The effect sizes (ES) were compared using Cohen's *d*.

**Results:**

The response rate was 82% and 4,683 (79.1%) had complete data available for analysis. Physical and Mental Component Summary (PCS and MCS) scores decreased as the number of chronic conditions increased monotonically (p<0.0001). Diabetes and hypertension negatively influenced PCS (mean score differences −0.6 and −1.5, p<0.001 respectively) but not MCS, whereas awareness of diabetes and hypertension negatively influenced MCS (−2.9 and −1.6, p<0.005 respectively) but not PCS. Arthritis had the largest ES on PCS (−0.37), while awareness of diabetes had the largest ES on MCS (−0.36). CVD moderately affected PCS and MCS (ES −0.34 and −0.27 respectively). Obesity had a negative effect on PCS (ES −0.27). Exercise positively affected PCS and MCS (ES +0.08 and +0.21 (p<0.01) respectively).

**Conclusion:**

Health promotion to reduce the prevalence of chronic diseases is important to improve the quality of life in Asian populations. Physical activity is an important part of such programs. Awareness of diseases may have greater impacts on mental health than having the disease itself. This has implications for the evaluation of the cost-benefit of screening and labeling of individuals with pre-disease states.

## Introduction

Health-Related Quality of Life (HRQoL) refers to the physical, emotional, and social impact of disease and treatments and is distinct from physiologic measures of disease. Increasingly, this measure has been used in clinical studies of patients with chronic conditions [1]. Studies have shown that living with a chronic condition significantly reduces HRQoL [2]–[10], and having multiple chronic conditions reduces HRQoL even further [11]–[13].

Hypertension and diabetes are two common conditions that are responsible for a considerable burden of chronic illness and death. In recent times, both have had new extended definitions to include so-called pre-hypertension and pre-diabetes states. But, at least for hypertension, those aware of their hypertension have been found to have a lower HRQoL than those unaware (i.e. there is a “labeling effect”) [14]–[18]. However, the impact of awareness of diabetes on HRQoL has been rarely explored, with two studies reporting that screening for diabetes has no impact on HRQoL or anxiety/depression [19], [20] and a further study finding higher levels of short-term anxiety among those diagnosed with diabetes compared to those who were not [21]. Moreover, HRQoL is also affected by lifestyle factors. With many factors affecting HRQoL, an understanding of their relative impact can assist in developing strategies aimed at improving HRQoL.

Whilst there have been many studies from the West, few studies have been conducted in Asia, where social norms, cultural and religious beliefs are different and may affect a person's perception of HRQoL. In this report, we study HRQoL in Thailand, examining the effects of lifestyle factors and different health states, including hypertension and diabetes, on HRQoL and comparing them to each other.

## Methods

The Electricity Generating Authority of Thailand (EGAT) study [22] is a longitudinal study comprising of 3 waves of recruitments, referred to as EGAT 1, 2 and 3. Data used in this study is cross-sectional, comprising data from the third EGAT 2 survey in 2008 and the first EGAT 3 survey in 2009: the most recent survey data, to date. From 5,915 invitees aged 25–70 years, 4,850 (82%) agreed to participate: 4000 working at the EGAT headquarters in Bangkok and 850 at hydro-electric plants in remote areas. Information was collected, through interviews by trained professionals, on age, sex, socioeconomic status (SES) and lifestyle, as well as details of self-reported common chronic medical conditions, including drug treatment. These self-reported conditions were coronary heart disease (CHD), stroke, peripheral arterial disease (PAD), chronic heart failure (CHF), diabetes, chronic kidney disease (CKD), liver disease, asthma, arthritis and rheumatism, systemic lupus erythematosus (SLE), Parkinson's disease, and epilepsy. CVD was defined as any of CHD, stroke, PAD and CHF.

HRQoL was measured using a bilingual version of the Short Form 36 (SF-36) version 2 questionnaire, which has been shown to be reliable and valid in Thailand [23]. The SF-36 measures eight domains (dimensions) of health status: physical functioning (PF), role-physical (RP), bodily pain (BP), general health (GH), vitality (VT), social functioning (SF), role-emotional (RE) and mental health (MH) [24]. SF-36 domain scores were further summarized into a physical component summary (PCS) score and a mental component summary (MCS) score. All scores range from zero (worst health) to 100 (best health), and are scaled relative to the United States population (mean = 50, standard deviation = 10).

Body mass index (BMI) was computed as measured weight in kilograms divided by the square of height in meters. Blood pressure was measured twice with an automatic device in the seated position, after five minutes rest and averaged. Hypertension was defined as systolic blood pressure (SBP) ≥140 mm Hg and/or diastolic blood pressure (DBP) ≥90 mm Hg, and/or self-reported (pharmacological) treatment for hypertension within the 2 weeks prior to the interview. Awareness of hypertension was defined as a self-report of any prior diagnosis of hypertension by a healthcare professional. Control of hypertension was defined as having an average SBP <140 mmHg and an average DBP <90 mmHg (in those not having diabetes and CKD) and an average SBP <130 mmHg and an average DBP <80 (in those with diabetes or CKD) in the context of pharmacological treatment of hypertension [25]. Diabetes was defined as an overnight fasting blood glucose ≥7.0 mmol/l and/or self-reported diabetes prior to the survey [26]. Control of diabetes was defined as having fasting blood glucose less than 126 mg/dl. As with hypertension, treatment for diabetes was self-reported and restricted to pharmacological treatment.

The Institutional Review Board at Mahidol University approved the study. Written informed consent was obtained from each participant before data were collected.

### Data analysis

Mean PCS and MCS scores were compared by age categories (sex adjusted), sex (age adjusted), SES and lifestyle variables (age and sex adjusted) and each self-reported chronic condition and number of chronic conditions (age, sex and SES adjusted) using generalized linear models (GLM) [27]. A multiple regression model was used to test for linear trend if a variable contained more than two categories. According to awareness, treatment and control of hypertension and diabetes, GLMs with full adjustment were used to evaluate the differences between no disease/disease, unaware/aware, untreated/treated and uncontrolled/controlled. Two sensitivity analyses were performed for awareness: (i) using the cut-off at 140/90 mmHg to determine controlled hypertension in all subjects (including subjects with CKD and diabetes) and (ii) taking comorbidity into the multivariable model. The effect sizes (ES), for PCS and MCS scores, were computed from all analyses on lifestyles, self-reported conditions and awareness, treatment and control of diseases, using Cohen's *d* [28]. The effects of those factors studied on HRQoL were of a similar magnitude and in the same direction for both sexes (selected results are shown in Appendix S1, S2), and so the sexes were combined. Results associated with a probability of ≤0.05 (two-tailed test) were considered statistically significant. Statistical analyses were performed using SAS version 9.2 (SAS Institute Inc., Cary, NC, USA).

## Results

### Summary statistics for SF-36

Of the 4,850 participants, 4,683 (97%) had complete information on SF-36 PCS and MCS scores and sociodemographic data, and were included in this study. Table 1 shows descriptive statistics of SF-36 scores for all participants. The mean PCS score was 49.6 and the MCS score was 50.8.

**Table 1:** Descriptive statistics for SF-36 scores (n = 4,683).

SF-36 component (and abbreviation)		No. of items	Range	Mean (SD)	Median (IQR)
Physical Functioning	(PF)	10	0–100	77.3 (19.4)	80 (65–95)
Role limitations due to Physical health	(RP)	4	0–100	86.7 (17.5)	94 (75–100)
Bodily Pain	(BP)	2	0–100	75.1 (21.2)	74 (62–100)
General Health	(GH)	5	0–100	63.2 (18.4)	65 (52–77)
Vitality	(VT)	4	6.25–100	66.9 (14.8)	69 (56–75)
Social Functioning	(SF)	2	12.5–100	84.4 (16.9)	88 (75–100)
Role limitations due to Mental health	(RE)	3	8.3–100	87.4 (17.3)	100 (75–100)
Mental Health	(MH)	5	5–100	74.6 (14.9)	75 (65–85)
**Physical Component Summary**	**(PCS)**	21	8.75–100	76.1 (13.9)	78 (68–87)
**Mental Component Summary**	**(MCS)**	14	22.7–100	78.3 (12.8)	80 (70–88)

Note: PF = physical functioning; RP = role physical; BP = bodily pain; GH = general health; VT = vitality; SF = social functioning; RE = role emotional; MH = mental health; PCS = physical component score (comprises of PF+RP+BP+GH); MCS = mental component score (comprises of VT+SF+RE+MH); SD = standard deviation; IQR = interquartile range (25^th^–75^th^ percentile).

SF-36 scores range from zero (worst health) to 100 (best health) and are scaled relative to those of the United States population (mean = 50, standard deviation = 10).

### Sociodemographic and lifestyle associations

Men had higher PCS and MCS scores than women (50.1 versus 48.3 and 51.0 versus 50.1 respectively, p<0.001). (Table 2) Older age and living in a rural area were associated with lower PCS but higher MCS, whereas higher education was associated with higher PCS but lower MCS (all p values <0.05). Exercise was associated with higher mean scores for both summary components (PCS 50.0 versus 49.4, p 0.008 and MCS 51.9 versus 50.2, P<0.0001). Increasing BMI was associated with lower PCS (p for trend <0.001) while it had no association with MCS.

**Table 2:** PCS and MCS norm-based scores according to socio-demographic and lifestyle characteristics (n = 4,683).

			Age and sex adjusted mean (SE)	
		n (%)	PCS	MCS
Sex	Male	3390 (72%)	50.1 (0.1)	51.0 (0.1)
	Female	1293 (28%)	48.3 (0.2)	50.1 (0.2)
			*p<0.001*	*p<0.001*
Age Group (years)	25–34	464 (10%)	52.5 (0.3)	49.6 (0.4)
	35–44	1257 (27%)	50.7 (0.2)	49.6 (0.2)
	45–54	2255 (48%)	48.9 (0.1)	51.0 (0.2)
	55–70	707 (15%)	47.7 (0.3)	53.0 (0.3)
			*p<0.001*	*p<0.001*
Rurality	Urban	3581 (76%)	49.7 (0.1)	50.6 (0.1)
	Rural	1102 (24%)	49.1 (0.2)	51.2 (0.2)
			*p* = *0.01*	*p* = *0.04*
Marital Status	Married	3526 (75%)	50.1 (0.2)	51.0 (0.2)
	Not married	1157 (25%)	49.4 (0.1)	50.7 (0.1)
			*p* = *0.01*	*p* = *0.2*
Education	Compulsory	602 (13%)	48.5 (0.3)	51.6 (0.3)
	Vocational	1323 (28%)	48.9 (0.2)	51.0 (0.2)
	Bachelor	2030 (43%)	49.9 (0.2)	50.4 (0.2)
	Master/Doctorate	728 (16%)	51.0 (0.3)	50.7 (0.3)
			*p<0.001*	*p* = *0.01*
Income (Baht/month)	<20,000	300 (6%)	48.8 (0.4)	51.1 (0.5)
	20,000–50,000	1576 (34%)	49.1 (0.2)	50.8 (0.2)
	50,000–100,000	2031 (43%)	49.7 (0.2)	50.5 (0.2)
	>100,000	776 (17%)	50.6 (0.2)	51.1 (0.3)
			*p<0.001*	*p* = *0.9*
Smoking	Never smoker	2800 (62%)	49.6 (0.1)	50.9 (0.2)
	Previous smoker	871 (20%)	49.6 (0.2)	50.6 (0.3)
	Current smoker	819 (18%)	49.3 (0.2)	50.6 (0.3)
			*p* = *0.2*	*p* = *0.4*
Current alcohol	No	1900 (41%)	49.5 (0.2)	51.1 (0.2)
	Yes	2763 (59%)	49.6 (0.1)	50.6 (0.2)
			*p* = *0.6*	*p* = *0.03*
Exercise	<3 sessions/week	3175 (68%)	49.4 (0.1)	50.2 (0.1)
	≥3 sessions/week	1491 (32%)	50.0 (0.2)	51.9 (0.2)
			*p* = *0.004*	*p<0.001*
Obesity as measured by BMI*	Underweight	167 (4%)	50.7 (0.5)	50.4 (0.6)
	Normal	2717 (58%)	50.1 (0.1)	50.7 (0.2)
	Overweight	1433 (31%)	49.0 (0.2)	50.8 (0.2)
	Obesity	344 (7%)	48.0 (0.4)	51.3 (0.4)
			*p<0.001*	*p* = *0.2*

Note: PCS = physical component score; MCS = mental component score; SF-36 scores range from zero (worst health) to 100 (best health) and are scaled relative to those of the United States population; p values for trend in variables with more than 2 categories;

* Body mass index (BMI): Underweight: <18.5 kg/m^2^; Normal: 18.5–24.9 kg/m^2^; Overweight: 25–29.9 kg/m^2^; Obesity: ≥30 kg/m^2^. SE = standard error.

### Self-reported disease

**Figure 1:**
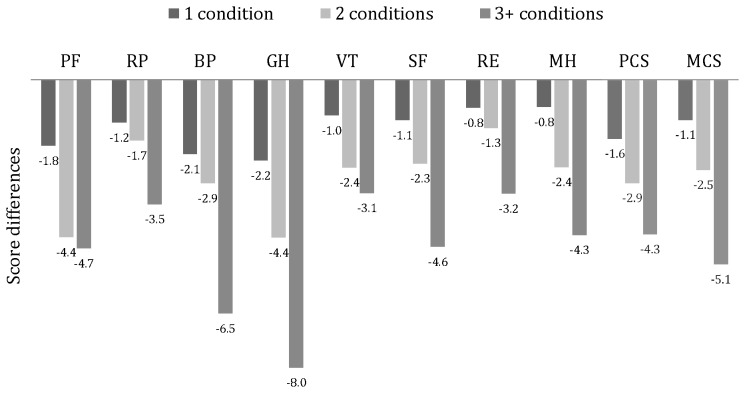
Magnitude of mean reductions in HRQoL in participants reporting one, two and three or more conditions; compared to subjects reporting no chronic medical condition. **Footnote**: PF = physical functioning; RP = role physical; BP = bodily pain; GH = general health; VT = vitality; SF = social functioning; RE = role emotional; MH = mental health; PCS = physical component score (comprises of PF+RP+BP+GH); MCS = mental component score (comprises of VT+SF+RE+MH); Chronic medical conditions includes 1.coronary heart disease (n = 70 cases) 2.congestive heart failure (n = 10) 3.stroke (n = 57) 4.peripheral arterial disease (n = 41) 5.chronic kidney disease (n = 65) 6.chronic liver disease (n = 532) 7.asthma (n = 224) 8.arthritis and rheumatism (n = 488) 9.diabetes mellitus (n = 315) 10.Parkinson's disease (n = 2) 11.epilepsy (n = 34) and 12.systemic lupus erythematosus (n = 33); Number of chronic medical conditions are determined by summing the conditions described above. All analyses were adjusted for age, sex, marital status, education, income and rurality. *All analyses yielded p for trend <0.0001.

Of the 12 health conditions that were assessed, 68% of participants reported having none, 25% reported 1 condition, 5.2% reported 2 conditions and 1.2% reported 3 or more conditions (to a maximum of 7). Chronic liver disease had the highest prevalence in this population, at 11.4%, followed by arthritis (10.4%), diabetes (6.7%), asthma (4.8%), CVD (3.4%) and CKD (1.4%). When adjusted for age, sex and SES, participants who were free from chronic conditions scored the highest HRQoL in all dimensions, and thus on PCS and MCS. Figure 1 displays the adjusted differences in mean SF-36 scores between those who were free from chronic conditions (the reference group) and those having 1, 2 or ≥3 chronic conditions. The differences between ≥3 and the reference were highest in GH (8.0) (p for trend <0.0001). Both PCS and MCS decreased when the number of chronic conditions increased, starting from 50.3 and 51.7 in the group with no chronic condition to 46.0 and 46.6 for those with ≥3 conditions (PCS and MCS respectively, p for trend <0.0001).

Table 3 shows specific results for the 5 most common conditions, excluding diabetes (see table 4 for the associations with diabetes). All but asthma negatively affected PCS (p<0.05), and all but CKD negatively affected MCS (p<0.05). Participants who reported having liver disease, CVD or arthritis had significantly lower SF-36 scores in every dimension, when compared to those who answered no for that specific disease (all p<0.05). Asthma was associated with reduced GH (p<0.05) while CKD was associated with reduced BP (p<0.05). PCS was lowest in those with arthritis and CVD (47.4 in both). MCS was lowest in CVD (49.1). The magnitude of the impacts of different diseases on HRQoL increased when compared to an alternative reference group comprising those without *any* reported chronic condition, rather than those without only the index disease. Percent distribution of comorbidity within these diseases is demonstrated in Appendix S3. Liver disease, asthma and arthritis share a similar disease pattern in that 66–67% of them are stand-alone conditions, followed by 25–26% with one accompanying condition and 7–8% with more than one condition. Multiple comorbidities are likely to be found in CVD and CKD, in that 43–46% of them have at least one accompanying condition. A sensitivity analysis in which we take number of comorbidity into account, gives grossly similar effects and statistical significances in each condition.

**Table 3:** Adjusted mean (with standard error) SF-36 norm-based scores according to absence or presence of the 5 most common self-reported chronic conditions *(with results for those with no reported chronic condition included for reference).

	No reported disease	Liver disease (n = 532)		Cardiovascular disease (n = 160)		Asthma (n = 224)		Arthritis & Rheumatism (n = 488)		Chronic kidney disease (n = 65)	
	(n = 3189)	Absent	Present	Absent	Present	Absent	Present	Absent	Present	Absent	Present
PF	48.2 (0.2)	47.6 (0.2)	45.7 (0.4)	47.5 (0.2)	45.3 (0.7)	47.4 (0.2)	46.5 (0.6)	47.8 (0.2)	44.5 (0.4)	47.4 (0.2)	45.7 (1.0)
RP	52.2 (0.1)	51.9 (0.1)	50.8 (0.3)	51.9 (0.1)	49.8 (0.4)	51.8 (0.1)	51.6 (0.4)	52.0 (0.1)	50.7 (0.3)	51.8 (0.1)	50.8 (0.7)
BP	51.4 (0.2)	50.7 (0.2)	48.9 (0.4)	50.6 (0.2)	48.2 (0.7)	50.6 (0.2)	50.0 (0.6)	51.0 (0.2)	47.1 (0.4)	50.6 (0.2)	48.3 (1.1)
GH	47.1 (0.2)	46.5 (0.2)	44.3 (0.4)	46.4 (0.2)	42.7 (0.7)	46.4 (0.2)	44.0 (0.6)	46.5 (0.2)	43.9 (0.5)	46.3 (0.2)	45.4 (1.1)
VT	53.8 (0.2)	53.5 (0.2)	52.0 (0.3)	53.4 (0.2)	52.0 (0.6)	53.4 (0.2)	52.6 (0.5)	53.5 (0.2)	52.4 (0.4)	53.4 (0.2)	53.3 (0.9)
SF	51.1 (0.2)	50.7 (0.2)	49.0 (0.4)	50.6 (0.2)	48.0 (0.6)	50.6 (0.2)	49.6 (0.5)	50.7 (0.2)	49.5 (0.4)	50.6 (0.2)	49.0 (0.9)
RE	52.2 (0.1)	52.0 (0.1)	51.1 (0.3)	52.0 (0.1)	49.8 (0.4)	51.9 (0.1)	51.8 (0.4)	52.0 (0.1)	51.2 (0.3)	51.9 (0.1)	51.6 (0.7)
MH	50.7 (0.2)	50.4 (0.2)	49.0 (0.4)	50.3 (0.2)	48.7 (0.7)	50.3 (0.2)	49.7 (0.6)	50.4 (0.2)	49.1 (0.4)	50.3 (0.2)	49.3 (1.0)
PCS	50.3 (0.2)	49.9 (0.2)	48.4 (0.3)	49.8 (0.2)	47.3 (0.6)	49.7 (0.2)	49.4 (0.5)	50.0 (0.2)	47.4 (0.5)	49.8 (0.2)	48.0 (0.9)
MCS	51.7 (0.2)	51.4 (0.2)	49.7 (0.4)	51.3 (0.2)	49.1 (0.7)	51.3 (0.2)	50.1 (0.6)	51.3 (0.2)	50.1 (0.4)	51.2 (0.2)	50.6 (1.0)

Note: PF = physical functioning; RP = role physical; BP = bodily pain; GH = general health; VT = vitality; SF = social functioning; RE = role emotional; MH = mental health; PCS = physical component score; MCS = mental component score;

Cardiovascular disease includes coronary heart disease, congestive heart failure, stroke and peripheral arterial disease.

SF-36 scores range from zero (worst health) to 100 (best health) and are scaled relative to those of the United States population (mean = 50, standard deviation = 10)

All analyses were adjusted for age, sex, marital status, education, income and rurality.

* Excluding diabetes: see table 4.

**Table 4:** Adjusted mean (with standard error) SF-36 norm-based scores according to hypertension and diabetes, and to awareness, drug treatment and control of hypertension and diabetes.

HYPERTENSION: Awareness, Treatment, Control				PCS	MCS
No disease (n = 3247)				49.5 (0.2)	51.1 (0.2)
Disease (n = 1436)				48.8 (0.2)	51.0 (0.3)
				*p* = 0.006	*p* = 0.6
	Unaware (n = 637)			48.1 (0.4)	51.9 (0.4)
	Aware (n = 799)			48.2 (0.4)	50.3 (0.4)
				*p* = 0.9	*p* = 0.0002
		Untreated (n = 152)		48.0 (0.7)	50.5 (0.8)
		Treated (n = 647)		47.3 (0.4)	50.2 (0.5)
				*p* = 0.4	*p* = 0.7
			Uncontrolled (n = 377)	46.8 (0.6)	49.9 (0.6)
			Controlled (n = 270)	47.7 (0.6)	50.4 (0.6)
				*p* = 0.1	*p* = 0.4

DIABETES: Awareness, Treatment, Control				PCS	MCS
No disease (n = 4262)				49.4 (0.2)	51.2 (0.2)
Disease (n = 420)				47.9 (0.4)	50.4 (0.4)
				*p*<0.0001	*p* = 0.08
	Unaware (n = 104)			47.4 (1.1)	52.1 (1.1)
	Aware (n = 316)			46.3 (0.8)	49.4 (0.8)
				*p* = 0.2	*p* = 0.003
		Untreated (n = 89)		46.6 (1.2)	49.6 (1.2)
		Treated (n = 227)		46.3 (0.9)	49.2 (0.9)
				*p* = 0.8	*p* = 0.6
			Uncontrolled (n = 146)	46.7 (1.1)	49.4 (1.0)
			Controlled (n = 81)	46.1 (1.2)	48.9 (1.2)
				*p* = 0.7	*p* = 0.7

Note: PCS = physical component score; MCS = mental component score;

SF-36 scores range from zero (worst health) to 100 (best health) and are scaled relative to those of the United States population (mean = 50, standard deviation = 10).

All analyses were adjusted for age, sex, marital status, education, income and rurality.

### Impact of awareness and treatment in people with hypertension and diabetes

Table 4 explores the associations between awareness, treatment and control of hypertension and diabetes and the SF-36 scores. Participants who had diabetes rated their PCS lower than non-diabetes (p<0.0001). The MCS scores were not different between having and not having this condition (p = 0.08). However, significant differences in MCS were found amongst those who were aware or not aware of having diabetes. Awareness of diabetes had a substantial negative impact on MCS (p = 0.003); conversely, awareness of diabetes did not influence the PCS score (p = 0.2). No difference in PCS and MCS were detected between treated and untreated; or controlled and uncontrolled groups. The impacts of awareness, treatment and control of hypertension on HRQoL displayed a similar picture to diabetes. The sensitivity analyses yielded similar effects and statistical significances in both conditions.

### Impact of disease severity in people who were aware of their disease

Appendix S4 shows adjusted SF-36 scores, according to severity of hypertension, diabetes, CKD and subgroups of CVD, in those who were aware of that particular disease. No relationship between disease severity and HRQoL was detected among subjects with hypertension, diabetes and CKD. There are significant differences in PCS and MCS amongst CVD subtypes.

### Effect sizes

**Figure 2:**
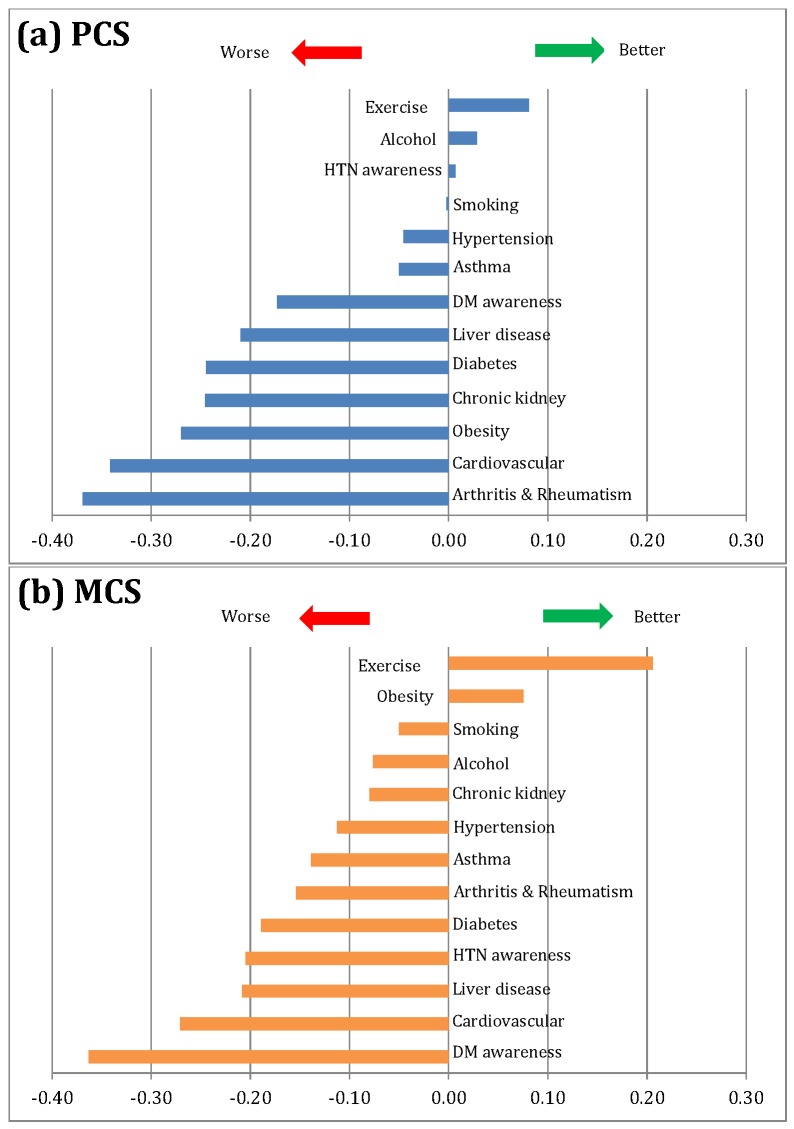
Effect Sizes* in the presence and absence of self-reported chronic conditions and lifestyles. A: Effect sizes on Physical Component Summary (PCS) score. B: Effect sizes on Mental Component Summary (MCS) score. **Footnote:** PCS = physical component score; MCS = mental component score; HTN = hypertension; DM = diabetes; Exercise (≥3 sessions/week vs <3/week); obesity (obesity vs normal); smoker and alcohol (current users vs no); all diseases are self-reported (yes vs no); awareness (previously diagnosed vs undiagnosed); For simplicity only presence/absence and treated/untreated disease categorization and only the most common 5 diseases are shown; All analyses were adjusted for age, sex, marital status, education, income and rurality. *Difference in means divided by the pooled standard deviation.

Taking all factors considered, arthritis had the largest negative impact (ES of −0.37) on PCS, followed by CVD (−0.34), obesity (−0.27), and CKD (−0.27) (figure 2). Awareness of diabetes and hypertension had smaller negative effects on PCS, compared to having a serious medical condition. Regarding the MCS, awareness of diabetes showed the greatest negative effect on MCS (−0.36), followed by CVD (−0.27), chronic liver disease (−0.21) and awareness of hypertension (−0.20). Exercise was the only factor to show a positive effect on both PCS (+0.08) and MCS (+0.21). These effects were comparable in size, but in the opposite direction, to having liver disease or diabetes. Lastly, obesity showed a trivial positive effect on MCS (+0.08), but a large negative effect on PCS (−0.27).

## Discussion

In addition to adding to the limited information on HRQoL in Asia, there are, as far as we are aware, two novel aspects to this study. This is the first time that the effects of multiple chronic conditions, awareness of diseases and lifestyle factors on HRQoL have been explicitly illustrated and made comparable to each other. Second, this study is the first to show that awareness of diabetes has a particularly strong impact on mental health, larger than having CVD or chronic liver disease. Other major findings are that exercise was the only factor considered that had a significant beneficial effect on both physical and mental health. Furthermore, its impact on mental health was of a sufficient magnitude to cancel out the adverse effects of most chronic conditions, at least in statistical terms. Obesity has a considerably large negative impact on physical health. These latter findings are particularly important since they involve readily modifiable factors.

The first use of the SF-36 in Thais was in 2000, when it was validated in Thai cardiac patients [29]. Since then the Thai version of SF-36 has been utilized in many clinical settings [8], [30], [31], as well as in a general population [23]. The relationship between the number of chronic conditions and HRQoL found here is consistent with other studies that found a trend towards poorer HRQoL when comorbidity increased [11]–[13]. In those having three or more conditions, four to eight point decrements in the domain scores and the biggest and smallest drops in GH and RE respectively, were similar here to those reported in Australia [13]. It is plausible that the wider gap in GH and BP was due to the selection of diseases that primarily affected physical, not mental, well-being.

Regarding awareness of hypertension: to date, there have been two published articles analyzing the impact of awareness of hypertension on HRQoL in adults, using the SF-36 instrument [14], [16]. Unlike our findings, both of these studies found that hypertension awareness predominately affected physical, rather than mental domains of HRQoL. A population-based study from Spain [14] reported significant reductions in PF, BP, VT and MH in known hypertensives compared to those who had hypertension but were not aware of it. However, the reductions in PCS and MCS scores were not significant in this study, possibly due to the small sample size (including only 104 hypertensive subjects) and the low response rate (31%). Another study found similar reductions only in PF and BP [16]. However, in that study the eligible participants had to have at least one CV risk factor; as a consequence, HRQoL might already be reduced in normotensive subjects. Our finding supports previous research linking the labeling effect of hypertension to psychological well-being [32]. Recent data from the US also showed that individuals labelled as hypertensive tend to self-report relatively poor health [17], [18]. Lastly, in the context of hypertension, our result is concordant with the well-established result that hypertensives have lower HRQoL compared to normal subjects [33].

Our study is the first to demonstrate the impact of diabetic awareness on HRQoL in Asia. Worldwide, there have been several studies on factors influencing HRQoL in diabetic patients [34]–[36], but few report the impact of diabetes screening on HRQoL [19]–[21]. Two of these reported a similar SF-36 score between those unaware that they had diabetes and subjects without diabetes [19], [20]. Another study found that subjects unaware of their diabetes had a higher anxiety score than subjects without diabetes, although the SF-36 was not administered in this study [21]. The high impact of awareness of diabetes on mental health found in this population might be due to individual's perception of the disease. In Thailand, where the standard of health care service is not yet comparable to that in Western countries, being labeled with diabetes could easily compromise mental health, especially in those socioeconomic disadvantaged. Diabetes itself exerts its detrimental effect on quality of life via several mechanisms; largely from its complications [34] and comorbidities [37]. In our analysis, those with established disease had the lowest physical health score followed by those unaware and those with no disease, respectively; however, this trend was not observed in the mental component. Instead, those who were unaware of diabetes had a marginally better mental health than those without diabetes (p = 0.08). Possibly this is a chance finding, although we cannot rule out “reverse causality” whereby, amongst people with diabetes, those with good mental health are more likely to seek medical help and thus become diagnosed.

While arthritis exhibited the greatest impact on physical function and pain, its impact on mental health was less than for awareness of hypertension. CVD, on the other hand, had a moderately large impact on both physical and mental health. The effects of CVD on general health, role limitations, and social functioning were greatest of all health conditions reported in this study. This finding confirmed the importance of CVD as a leading global disease burden. The impact of obesity on physical health was only secondary to CVD and arthritis, and was larger than CKD, diabetes or liver disease. Previous studies in the UK [38] and Sweden [39] also reported decreasing PCS with obesity. Although obesity showed a positive effect on mental health, this difference did not reach a statistical significance, and was consistent with previous research in Asian populations [40]–[43]. This may be explained by Oriental social and cultural norms, where moderate overweight is related to wealth and happiness.

The positive effect of exercise on HRQoL was consistent with findings from a systematic review [44]. Although we cannot determine whether our association can be explained via a causal pathway, research has proved the benefits of exercise on health. Further, exercise, as a lifestyle intervention, has been implemented into several clinical practice guidelines [45]–[48]. Here, we extend knowledge by comparing the effect of exercise to other conditions. The positive effect of exercise on mental health was comparable in size to detrimental effects of chronic liver disease, and was larger than the effects of arthritis, asthma or diabetes. One study showed that a programme of exercise increased quality of life in diabetic patients [49]. Whether exercise can attenuate the detrimental effects of other chronic medical conditions on HRQoL needs further research. This study has the advantages of a large sample size and the inclusion of several variables which allowed us to compare the adjusted effect sizes of multiple health states. Nevertheless, there are several limitations. First, the healthy worker effect may apply: those having significant underlying health problems may not have entered the workforce or participated in the survey. This may dilute the magnitude of impacts of poor health on HRQoL, but is less likely to influence the relative effects of different lifestyle, and other, factors. Second, details on malignancy and mental illness are lacking in this study. However, we did cover a large number of other important chronic conditions. Third, data on awareness, treatment and control was only collected for diabetes and hypertension. Forth, measuring HRQoL by a general health questionnaire might not be as sensitive to detect a problem as using a disease specific questionnaire. However in a population context, especially when we are comparing different diseases, the application of a non-specific tool is more practical. Finally, this is a cross sectional study and as such, it is difficult to be certain of the direction of causality. However, our conclusions about diabetes awareness on HRQoL are supported by data from randomized controlled trials [21].

In conclusion, despite a different socio-ethnic background, the impact on HRQoL of several key chronic health states, lifestyle factors and disease awareness in an urban Thai population were similar to those previously seen in Western populations. Many of the chronic conditions studied are common, worldwide, and thus health promotion is an important part of improving the quality of life of a population. Being physically active was associated with better physical and mental health and this is an important part of such interventions. Awareness of diabetes and hypertension was associated with impaired mental health, with a magnitude greater than having the disease itself. This suggests that awareness is an important consideration when evaluating the cost-benefit of screening programs and also when employing labels such as pre-hypertension and pre-diabetes [50]. Further evaluation using prospective study designs is required to establish causality.

## Supporting Information

Appendix S1 Age adjusted mean (with standard error) PCS and MCS norm-based scores according to socio-demographic and lifestyle characteristics by sex.
(DOCX)

Appendix S2 Adjusted mean (with Standard Error) SF-36 norm-based scores according to number of chronic condition and by absence or presence of the 6 most common self-reported chronic conditions by sex.
(DOCX)

Appendix S3 Percent distribution of comorbidity within top 6 self-reported condition.
(DOCX)

Appendix S4 Adjusted mean (with Standard Error) SF-36 norm-based scores according to severity of hypertension, diabetes, chronic kidney disease and subgroups of cardiovascular disease, in those who were aware of their disease.
(DOCX)
