# Supplementary material for: Associations of Lifestyle Factors, Disease History and Awareness with Health-Related Quality of Life in a Thai Population
Source: PLoS One. 2012 Nov 26;7(11):e49921. doi: 10.1371/journal.pone.0049921 (PMC3506606; doi:10.1371/journal.pone.0049921)
Supplement: Appendix S1 — Age adjusted mean (with standard error) PCS and MCS norm-based scores according to socio-demographic and lifestyle characteristics by sex. (DOCX) [file pone.0049921.s001.docx]

Appendix 1: Age adjusted mean (with standard error) PCS and MCS norm-based scores according to socio-demographic and lifestyle characteristics by sex

|  |  |  | Male | | Female | | |
| --- | --- | --- | --- | --- | --- | --- | --- |
|  |  | n | PCS | MCS | n | PCS | MCS |
| Age Group | 25-34 | 338 | 52.7 (5.8) | 49.8 (7.9) | 126 | 52.0 (5.6) | 49.0 (8.2) |
| (Years) | 35-44 | 884 | 50.9 (6.2) | 49.9 (7.6) | 373 | 50.2 (6.7) | 48.8 (8.7) |
|  | 45-54 | 1648 | 49.6 (7.0) | 51.1 (7.7) | 607 | 47.3 (7.2) | 50.6 (8.1) |
|  | 55-70 | 520 | 48.5 (7.1) | 53.5 (7.7) | 187 | 45.5 (8.2) | 51.4 (8.2) |
|  |  |  | <0.001 | <0.001 |  | <0.001 | <0.001 |
| Rurality | Urban | 2530 | 50.2 (6.7) | 50.8 (7.8) | 1051 | 48.8 (7.0) | 49.7 (8.5) |
|  | Rural | 860 | 49.4 (6.8) | 51.7 (7.7) | 242 | 46.3 (8.5) | 51.5 (7.7) |
|  |  |  | 0.001 | 0.6 |  | <0.001 | 0.002 |
| Marital Status | Married | 2719 | 49.7 (6.8) | 51.2 (7.8) | 807 | 48.0 (7.5) | 49.8 (8.3) |
|  | Not married | 671 | 51.5 (6.5) | 50.3 (7.8) | 486 | 48.9 (7.1) | 50.4 (8.5) |
|  |  |  | <0.001 | 0.003 |  | 0.03 | 0.3 |
| Education | Secondary | 486 | 48.1 (7.7) | 52.7 (8.2) | 116 | 46.4 (8.8) | 50.9 (8.1) |
|  | Vocational | 1061 | 49.4 (6.8) | 51.3 (7.6) | 262 | 46.1 (7.5) | 51.0 (7.8) |
|  | Bachelor | 1400 | 50.6 (6.4) | 50.4 (7.8) | 630 | 48.7 (7.0) | 49.7 (8.5) |
|  | Master/Doctorate | 443 | 52.0 (6.1) | 50.3 (7.5) | 285 | 50.5 (6.5) | 49.5 (8.5) |
|  |  |  | <0.001 | <0.001 |  | <0.001 | 0.02 |
| Income | <20,000 | 232 | 49.4 (7.4) | 51.6 (8.7) | 68 | 47.7 (7.9) | 49.8 (8.4) |
| (Baht/Month) | 20,000-50,000 | 1193 | 49.8 (7.0) | 50.7 (7.9) | 383 | 48.4 (7.5) | 50.2 (8.2) |
|  | 50,000-100,000 | 1490 | 50.2 (6.6) | 50.9 (7.6) | 541 | 48.1 (7.3) | 49.8 (8.3) |
|  | >100,000 | 475 | 50.8 (6.5) | 51.7 (7.7) | 301 | 49.0 (6.9) | 50.3 (8.5) |
|  |  |  | 0.002 | 0.3 |  | 0.2 | 0.8 |
| Smoking | Never smoker | 1663 | 50.4 (6.7) | 51.0 (7.9) | 1137 | 48.4 (7.4) | 50.0 (8.4) |
|  | Previous smoker | 806 | 49.8 (6.8) | 51.2 (7.4) | 65 | 47.3 (8.1) | 50.8 (7.8) |
|  | Current smoker | 766 | 49.6 (6.8) | 51.0 (8.1) | 53 | 48.7 (6.7) | 50.2 (8.7) |
|  |  |  | 0.002 | 0.9 |  | 0.9 | 0.6 |
| Alcohol | Yes | 2355 | 50.2 (6.7) | 50.9 (7.7) | 408 | 48.1 (7.4) | 49.2 (8.2) |
|  | No | 1022 | 49.8 (7.1) | 51.5 (8.0) | 878 | 48.9 (7.0) | 50.4 (8.4) |
|  |  |  | 0.2 | 0.03 |  | 0.08 | 0.02 |
| Exercise | < 3 sessions/week | 2264 | 50.0 (6.6) | 50.4 (7.8) | 911 | 48.3 (7.4) | 49.5 (8.6) |
|  | ≥ 3 sessions/week | 1113 | 50.2 (7.0) | 52.4 (7.6) | 378 | 48.3 (7.3) | 51.4 (7.7) |
|  |  |  | 0.6 | <0.001 |  | 1.0 | <0.001 |

Note: PCS=physical component score; MCS=mental component score; SF-36 scores range from zero (worst health) to 100 (best health) and are scaled relative to those of the United States population; p values for trend in variables with more than 2 categories;
