# Supplementary material for: Associations of Lifestyle Factors, Disease History and Awareness with Health-Related Quality of Life in a Thai Population
Source: PLoS One. 2012 Nov 26;7(11):e49921. doi: 10.1371/journal.pone.0049921 (PMC3506606; doi:10.1371/journal.pone.0049921)
Supplement: Appendix S2 — Adjusted mean (with Standard Error) SF-36 norm-based scores according to number of chronic condition and by absence or presence of the 6 most common self-reported chronic conditions by sex. (DOCX) [file pone.0049921.s002.docx]

Appendix 2: Adjusted mean (with Standard Error) SF-36 norm-based scores according to number of chronic condition and by absence or presence of the 6 most common self-reported chronic conditions by sex

|  | |  | Male | | | Female | | |
| --- | --- | --- | --- | --- | --- | --- | --- | --- |
|  | |  | n | PCS | MCS | n | PCS | MCS |
| Number of chronic medical conditions | | 0 | 2281 | 50.7 (0.14) | 51.5 (0.16) | 908 | 49.0 (0.23) | 50.4 (0.28) |
|  |  | 1 | 864 | 49.2 (0.23) | 50.3 (0.27) | 327 | 47.3 (0.41) | 49.6 (0.49) |
|  |  | 2 | 196 | 48.1 (0.45) | 49.0 (0.52) | 49 | 45.3 (0.81) | 48.2 (0.96) |
|  |  | 3+ | 49 | 46.5 (0.84) | 47.9 (0.97) | 9 | 44.3 (1.76) | 47.1 (2.08) |
|  | |  |  | <0.0001 | <0.0001 |  | <0.0001 | 0.007 |
|  | |  |  |  |  |  |  |  |
| Cardiovascular disease | | Present | 139 | 47.8 (0.56) | 49.0 (0.64) | 21 | 46.5 (1.54) | 48.5 (1.81) |
|  |  | Absent | 3251 | 50.2 (0.12) | 51.1 (0.13) | 1272 | 48.4 (0.20) | 50.1 (0.23) |
|  | |  |  | <0.0001 | 0.001 |  | 0.2 | 0.4 |
| Chronic kidney disease | | Present | 53 | 48.0 (0.92) | 50.4 (1.06) | 12 | 47.7 (2.04) | 49.4 (2.40) |
|  |  | Absent | 3337 | 50.1 (0.11) | 51.0 (0.13) | 1281 | 48.3 (0.20) | 50.1 (0.23) |
|  | |  |  | 0.02 | 0.6 |  | 0.8 | 0.8 |
| Asthma | | Present | 153 | 49.5 (0.54) | 49.8 (0.62) | 71 | 48.7 (0.84) | 49.3 (1.00) |
|  | | Absent | 3237 | 50.1 (0.12) | 51.1 (0.13) | 1222 | 48.3 (0.20) | 50.1 (0.23) |
|  | |  |  | 0.3 | 0.04 |  | 0.6 | 0.4 |
| Liver disease | | Present | 447 | 48.9 (0.31) | 49.6 (0.36) | 85 | 46.8 (0.77) | 48.1 (0.90) |
|  | | Absent | 2943 | 50.3 (0.12) | 51.2 (0.14) | 1208 | 48.4 (0.20) | 50.2 (0.24) |
|  | |  |  | <0.0001 | <0.0001 |  | 0.04 | 0.02 |
| Arthritis | | Present | 304 | 48.1 (0.38) | 50.0 (0.44) | 184 | 45.5 (0.53) | 49.0 (0.63) |
|  | | Absent | 3086 | 50.3 (0.12) | 51.1 (0.14) | 1109 | 48.8 (0.21) | 50.2 (0.25) |
|  | |  |  | <0.0001 | 0.01 |  | <0.0001 | 0.06 |
| Diabetes | | Present | 255 | 48.7 (0.45) | 50.1 (0.52) | 60 | 46.3 (0.96) | 48.9 (1.13) |
|  | | Absent | 3135 | 50.5 (0.19) | 51.5 (0.23) | 1233 | 48.4 (0.34) | 50.4 (0.40) |
|  | |  |  | <0.0001 | 0.007 |  | 0.03 | 0.2 |
| Obesity as measured by BMI | Underweight | | 72 | 50.7 (6.6) | 51.0 (8.0) | 95 | 51.1 (5.7) | 48.3 (9.4) |
|  | Normal | | 1918 | 50.7 (6.6) | 50.9 (7.9) | 799 | 48.7 (7.2) | 50.0 (8.3) |
|  | Overweight | | 1143 | 49.3 (7.1) | 51.2 (7.6) | 290 | 47.4 (7.4) | 50.2 (8.4) |
|  | Obesity | | 242 | 48.9 (6.7) | 51.5 (7.7) | 102 | 45.6 (8.4) | 51.2 (7.6) |
|  | |  |  | <0.001 | 0.2 |  | <0.001 | 0.05 |

Note: PCS=physical component score; MCS=mental component score; SF-36 scores range from zero (worst health) to 100 (best health) and are scaled relative to those of the United States population; p values for trend in variables with more than 2 categories; Body mass index (BMI): Underweight:<18.5kg/m^2^; Normal:18.5-24.9kg/m^2^; Overweight:25-29.9kg/m^2^; Obesity:≥30 kg/m^2^;

Cardiovascular disease includes coronary heart disease, congestive heart failure, stroke and peripheral arterial disease; all analyses were adjusted for age, marital status, education, income and rurality.
