# Supplementary material for: Associations of Lifestyle Factors, Disease History and Awareness with Health-Related Quality of Life in a Thai Population
Source: PLoS One. 2012 Nov 26;7(11):e49921. doi: 10.1371/journal.pone.0049921 (PMC3506606; doi:10.1371/journal.pone.0049921)
Supplement: Appendix S3 — Percent distribution of comorbidity within top 6 self-reported condition. (DOCX) [file pone.0049921.s003.docx]

Appendix 3: Percent distribution of comorbidity within top 6 self-reported condition

| Numbers of chronic conditions | Liver disease  (n=532) | Cardiovascular disease  (n=160) | Asthma  (n=224) | Arthritis & Rheumatism  (n=488) | Chronic kidney disease  (n=65) | Diabetes  (n=315) |
| --- | --- | --- | --- | --- | --- | --- |
| 1 condition  (n=1191) | 66%  (n=351) | 54%  (n=87) | 66%  (n=149) | 67%  (n=328) | 57%  (n=37) | 63%  (n=197) |
| 2 conditions  (n=245) | 26%  (n=139) | 27%  (n=43) | 26%  (n=59) | 25%  (n=121) | 23%  (n=15) | 27%  (n=86) |
| ≥3 conditions  (n=58) | 8%  (n=42) | 19%  (n=30) | 7%  (n=16) | 8%  (n=39) | 20%  (n=13) | 10%  (n=32) |

Chronic medical conditions includes 1.coronary heart disease 2.congestive heart failure 3.stroke 4.peripheral arterial disease 5.chronic kidney disease 6.chronic liver disease 7.asthma 8.arthritis and rheumatism 9.diabetes mellitus 10.Parkinson’s disease 11.epilepsy and 12.systemic lupus erythematosus. Numbers of chronic medical conditions are determined by summing the conditions described above.
