# Supplementary material for: Associations of Lifestyle Factors, Disease History and Awareness with Health-Related Quality of Life in a Thai Population
Source: PLoS One. 2012 Nov 26;7(11):e49921. doi: 10.1371/journal.pone.0049921 (PMC3506606; doi:10.1371/journal.pone.0049921)
Supplement: Appendix S4 — Adjusted mean (with Standard Error) SF-36 norm-based scores according to severity of hypertension, diabetes, chronic kidney disease and subgroups of cardiovascular disease, in those who were aware of their disease. (DOCX) [file pone.0049921.s004.docx]

Appendix 4: Adjusted mean (with Standard Error) SF-36 norm-based scores according to severity of hypertension, diabetes, chronic kidney disease and subgroups of cardiovascular disease, in those who were aware of their disease.

|  |  |  | Multivariable adjusted mean (SE) | |
| --- | --- | --- | --- | --- |
|  |  | n | PCS | MCS |
| Hypertension | |  |  |  |
|  | Controlled hypertension  (BP < 140/90 mmHg) | 318 | 47.6 (0.5) | 50.2 (0.6) |
|  | Hypertension, stage 1  (BP 140-159/90-99 mmHg) | 322 | 47.4 (0.5) | 50.2 (0.6) |
|  | Hypertension, stage 2  (BP ≥ 160/100 mmHg) | 159 | 47.3 (0.7) | 50.3 (0.7) |
|  |  |  | p 0.4 | p 0.9 |
| Diabetes | |  |  |  |
|  | Lifestyle modification | 86 | 46.6 (1.2) | 49.6 (1.2) |
|  | Oral hypoglycemic drugs | 197 | 46.2 (0.9) | 49.2 (0.9) |
|  | Insulin therapy | 33 | 46.7 (1.6) | 48.9 (1.6) |
|  |  |  | p 0.6 | p 0.5 |
| Chronic Kidney Disease | |  |  |  |
|  | Stage 1 | 55 | 46.5 (1.4) | 47.0 (1.8) |
|  | Stage 2-5 | 10 | 45.4 (2.2) | 48.5 (2.9) |
|  |  |  | p 0.6 | p 0.6 |
| Cardiovascular disease | |  |  |  |
|  | Coronary artery disease | 58 | 46.7 (0.9) | 50.1 (1.1) |
|  | Cerebrovascular disease | 50 | 47.3 (1.0) | 48.7 (1.2) |
|  | Peripheral arterial disease | 30 | 45.5 (1.2) | 47.8 (1.4) |
|  | Chronic heart failure | 6 | 47.2 (3.4) | 45.8 (3.9) |
|  | Poly-vascular disease | 16 | 47.2 (1.7) | 48.5 (2.0) |
|  |  |  | p 0.0002 | P 0.01 |

Note: PCS=physical component score; MCS=mental component score; SF-36 scores range from zero (worst health) to 100 (best health) and are scaled relative to those of the United States population; p values for trend for hypertension, diabetes and CKD; p values for difference between groups for CVD; all analyses were adjusted for age, marital status, education, income and rurality.
